# Preoperative serum calcium could be a prognostic factor for surgical treatment of recurrent patellar dislocation: a retrospective study

**DOI:** 10.1186/s12891-022-05527-y

**Published:** 2022-06-15

**Authors:** Yi Qiao, Zipeng Ye, Junjie Xu, Xiuyuan Zhang, Jiebo Chen, Caiqi Xu, Song Zhao, Jinzhong Zhao

**Affiliations:** Department of Sports Medicine, Shanghai Jiao Tong University Affiliated Sixth People’s Hospital, 600 Yishan Road, Shanghai, 200233 China

**Keywords:** Recurrent patellar dislocation, Prognosis, Calcium, Serum biomarker

## Abstract

**Background:**

Surgical treatment for recurrent patellar dislocation (RPD) could yield good outcomes. While, unsatisfactory recovery still exists in some cases. For all prognostic factors, serum biomarkers have rarely been investigated. This study aimed to evaluate the prognostic value of preoperative serum calcium level, a widely used serum biomarker, in surgical treatment for RPD.

**Study design:**

Retrospective study.

**Methods:**

Ninety-nine patients with RPD were enrolled in the study. Preoperative serum calcium was acquired from routinely tested blood 1 day prior to operation. Demographic data, characteristics of RPD, postoperative functional outcomes were obtained. The association between preoperative calcium and postoperative functional outcomes (Kujala, Lysholm, Tegner, IKDC and KOOS score) was determined by correlation analysis and multivariate linear regression analysis. Poor recovery was determined as Kujala score below 80. The receiver operating characteristic (ROC) curve was used to assess the prognostic value of preoperative calcium.

**Results:**

Patients were followed up for a mean period of 2.45 ± 1.33 years. All clinical scores showed significant improvement at the latest follow-up. Correlation and multivariate linear analyses indicated that serum calcium level was an important factor related with the prognosis of surgical treatment for RPD. According to the ROC curve, the cut-off value for preoperative calcium was 2.225 mmol/L. The clinical outcomes of patients with a preoperative blood calcium < 2.225 mmol/L was significantly worse than that with a higher calcium level. The correspondent sensitivity was 0.812 with a specificity of 0.633.

**Conclusion:**

Operative treatment for RPD achieved good results, while in some cases the functional scores remain inferior. As a serum biomarker, preoperative calcium could be prognostic for outcomes after surgical treatment for RPD.

## Introduction

Recurrent patellar dislocation (RPD), a common issue plaguing adolescent, is associated with various anatomical factors including soft tissue and bony abnormalities [1–3]. Medial patellofemoral ligament (MPFL) reconstruction combined with or without tibial tubercle (TT) transfer has been widely employed to treat recurrent patellar dislocation and usually yields favorable outcomes [4]. However, inferior postoperative outcomes are still presented in some cases and reoperation may be required [5].

Identification of prognostic factors may help classify patients for whom postoperative outcome is more likely to be inferior and pay special attention to improve these patients’ recovery [6]. Numerous factors have been investigated for recurrent patellar dislocation, including age, sex, patella alta, trochlear dysplasia, J-sign and various surgical techniques [7, 8]. Multiple researchers have developed predictive models for recurrent instability by combining these individual factors in different ways [9, 10]. To date, these models remain complex and require multiple imaging examinations, imposing a burden on patients’ cost. Postoperative recovery is affected by metabolic processes associated with inflammation [11], collagen or bone turnover [12], which may have a role in predicting the prognosis. Biomarkers, as a manifestation of these metabolic processes, have a potential utility in developing early diagnoses and evaluating prognosis [13]. Compared with other methods, biomarkers can be collected relatively easily form serum or urine and may be less costly. The identification of such biomarkers could better assist practitioners in assessing the prognosis of patients undergoing operation. As a widespread secondary messenger within multiple intracellular signaling pathways, calcium is involved in the bone turnover and a myriad of vital cellular activities including proliferation, motility and apoptosis, suggesting that calcium plays an important role in tissue regeneration and parenchymal function across the body [14]. In recent years, serum calcium level has been used to help determine the prognosis of cardiovascular event, cognitive decline and cancer [15–17].

Therefore, the aim of this study was to evaluate the association between preoperative serum calcium level and the clinical outcome after surgical treatment for recurrent patellar dislocation. Our assumption was that the serum calcium level could be a prognostic factor of recurrent patellar dislocation.

## Methods

### Study design and participants

This was a single-center, retrospective study. It was registered in the Chinese Clinical Trial Registry (ChiCTR2100052216) in 22/10/2021. The methods were performed in accordance with the relevant guidelines and regulations. We retrospectively studied the patients that were consecutively enrolled in the institutional database program of RPD from January 2017 to October 2020. The inclusion criteria were closed epiphysis, more than 2 episodes of dislocation or 1 episode of dislocation plus multiple episodes of instability (lateral excursion of the patella), and a tibial tubercle-trochlear groove (TT-TG) distance > 15 mm. The exclusion criteria were concomitant ligament injuries (except MPFL), previous knee surgeries, and femoral trochlear dysplasia requiring trochleoplasty.

### Surgical techniques and rehabilitation

A single experienced surgeon (J.Z.) in our institution conducted the surgical procedures. The whole procedure was similar to that described by Zhao et al. [18]. MPFL was reconstructed with autogenous semitendinosus tendons and by tunnel technique. Lateral retinaculum release was performed by pulling the patella medially with the graft. The procedure of TT transfer is a modification of Fulkerson’s osteotomy. The proximal part of the bone fragment was transferred anteromedially, with the distal tapering end left in place or, in some cases, rotated laterally along the osteotomy surface. The purpose was to mitigate the TT-TG distance to 5-10 mm.

Partial to full weightbearing was allowed with a hinged brace locked in extension immediately after the operation. The brace was removed at 6 weeks after surgery. Progressive range of motion exercises were started on the first day after surgery. Squats against the wall, step-up exercises and proprioception training were started at 6 weeks postoperatively. Running and agility training was permitted 3 months after the operation.

### Clinical variables and outcome measurements

Demographic data including sex, body mass index, age, and characteristics of patellar dislocation including patellar height, femoral trochlear dysplasia, lateral patellar tilt, sulcus angle, and TT-TG distance were recorded. Serum calcium level 1 day before the operation was also recorded. Functional scores were obtained preoperatively and at the latest follow-up, including the Kujala score, Knee Injury and Osteoarthritis Outcome (KOOS) score, Tegner Score, International Knee Documentation Committee (IKDC) subjective score and Lysholm score. Poor recovery was defined as a Kujala score below 80 [5].

### Statistical analysis

Analyses of the data obtained in the study were performed with SPSS (26.0; IBM). The Student t test or Mann-Whitney U test was conducted to compare the postoperative outcomes according to the normality of the data. The chi-square test was used to compare nominal variables. Pearson or Spearman correlation analysis was performed to analyze the relationship between obtained variables and postoperative outcomes. Multivariate linear regression analysis was conducted when more than one factor showed a significant correlation with 1 postoperative outcome. The predictive threshold value was determined by ROC curve and Youden index. Statistical significance was set at 0.05.

Since there are no previous reports testing the diagnosis value of serum calcium for postoperative recovery of patellar dislocation, a sample size calculation was not available prior to the patient enrollment. A post hoc calculation was instead performed with PASS software (15.0) according to the sensitivity and specificity acquired. The type I error was set at 0.05 with a permissible error of 10% for sensitivity and specificity.

## Results

Ninety-nine patients were included in this study. The baseline demographic data of the patients are provided in Table 1. No chronic effusion or other adverse events were reported after the surgery. All postoperative functional scores improved significantly compared with preoperative scores (Table 2).

**Table 1:** Descriptive statistics for patient demographics

	Values
Patients, n	99
Age, y	23.9 ± 6.8
Sex	
Female, n (%)	71 (71.7%)
Male, n (%)	28 (28.3%)
Body mass index	22.4 ± 4.4
Follow-up, y	2.45 ± 1.33
Side of patellar dislocation, n (%)	
Left	52 (52.5%)
Right	47 (47.5%)
Symptom duration, mo	56.7 ± 77.6
TT-TG distance, mm	19.3 ± 4.3
Dejour none/A/B/C/D	4/5/35/26/29
Insall-Salvati ratio	1.4 ± 0.4
Lateral patellar tilt, deg	30.6 ± 10.7
Sulcus angle, deg	153.7 ± 8.8
Serum calcium, mmol/L	2.29 ± 0.11

Data are presented as mean ± standard deviation unless otherwise noted

*TT-TG distance* tibial tubercle to trochlear groove distance

**Table 2:** Comparison between preoperative and postoperative outcomes

Outcome	Preoperative	Postoperative	*P* Value
Kujala score	67.2 ± 20.0	85.3 ± 14.3	< .001
Lysholm score	67.9 ± 17.5	85.5 ± 12.6	< .001
Tegner score	2.41 ± 0.93	4.00 ± 1.56	< .001
IKDC score	60.9 ± 15.9	75.1 ± 12.0	< .001
KOOS scores			
Pain	77.9 ± 15.0	92.5 ± 8.7	< .001
Symptoms	61.0 ± 14.8	70.4 ± 16.3	< .001
Daily living activities	77.3 ± 17.3	95.9 ± 5.7	< .001
Sports and recreation	52.0 ± 29.8	80.7 ± 18.6	< .001
Knee-related quality of life	44.1 ± 21.8	63.3 ± 21.0	< .001

Data are presented as mean ± standard deviation

*IKDC* International Knee Documentation Committee, *KOOS* Knee Injury and Osteoarthritis Outcome Score

The correlation between the serum calcium and postoperative results was analyzed (Table 3). Preoperative calcium was significantly and positively associated with postoperative Kujala score (*P* = .001), IKDC score (*P* < .001), KOOS pain score (*P* = .001), KOOS daily living activities score (*P* = .018), KOOS sports and recreation (*P* = .001), and KOOS knee-related quality of life (*P* = .035). These correlations suggested that a higher calcium level was associated with a better postoperative recovery.

**Table 3:** Correlation between preoperative calcium level and follow-up outcomes

Outcome	Correlation (*B*)	*P* Value
Kujala score	0.326	**.001**
Lysholm score	0.147	.147
Tegner score	0.177	.111
IKDC score	0.386	**< .001**
KOOS scores		
Pain	0.329	**.001**
Symptoms	0.101	.321
Daily living activities	0.238	**.018**
Sports and recreation	0.341	**.001**
Knee-related quality of life	0.212	**.035**

Significant *P* values are bolded

*IKDC* International Knee Documentation Committee, *KOOS* Knee Injury and Osteoarthritis Outcome Score

For other potentially critical factors, a correlation analysis was conducted (Table 4). An older age was significantly related to worse postoperative outcomes: IKDC score (B = − 0.297; *P* = .003), Lysholm score (B = − 0.316; *P* = .001), Kujala score (B = − 0.310; *P* = .002), KOOS pain score (B = − 0.385; *P* = .001), KOOS daily living activities score (B = − 0.309; *P* = .002), and KOOS sports score (B = − 0.227; *P* = .024). Additionally, a greater Insall-Salvati ratio was associated with a worse KOOS symptom score (B = − 0.231; *P* = .043) and KOOS knee-related quality of life (B = − 0.247; *P* = .030). A greater sulcus angle was associated with a worse KOOS pain score (B = − 0.225; *P* = .028).

**Table 4:** *P* values for correlation analyses between variables suspected to be relevant to postoperative outcomes

	IKDC	Lysholm	Kujala	KOOS-Symptom	KOOS-Pain	KOOS-ADL	KOOS-Sports	KOOS-QoL	Tegner
Sex	.159	.255	.623	.363	.860	.814	.546	.935	.157
Age	**.003** (− 0.297)	**.001** (− 0.316)	**.002** (− 0.310)	.158	**.001** (− 0.385)	**.002** (− 0.309)	**.024** (− 0.227)	.104	.076
BMI	.665	.380	.631	.333	.526	.123	.998	.957	.609
Symptom duration	.399	.504	.938	.127	.793	.650	.989	.305	.429
TT-TG distance	.130	.921	.473	.118	.875	.839	.619	.710	.896
Dejour type	.589	.724	.326	**.038** (0.210)	.353	.523	.362	.583	.996
Insall-Salvati ratio	.669	.453	.344	**.043** (−0.231)	.833	.448	.958	**.030** (−0.247)	.096
Lateral patellar tilt	1.000	.810	.288	.385	.312	.187	.604	.980	.407
Sulcus angle	.384	.061	.372	.546	**.028** (−0.225)	.165	.395	.994	.515

Significant *P* values are bolded

*IKDC* International Knee Documentation Committee, *KOOS* Knee Injury and Osteoarthritis Outcome Score, *ADL* activities of daily living, *QoL* quality of life

To further identify whether calcium or age was independently associated with postoperative recovery, multivariate linear regression analysis was performed (Table 5). It was indicated that age and calcium acted together in postoperative Kujala and KOOS pain score, while preoperative calcium was the only factor associated with IKDC (*P* = .001), KOOS sports (*P* = .004) and life quality score (*P* = .035).

**Table 5:** Multivariate linear regression analysis between clinical variables suspected to be relevant to postoperative outcomes and recovery

		IKDC	Kujala	KOOS-Pain	KOOS-ADL	KOOS-Sports	KOOS-QoL
Calcium	β	0.323	0.251	0.222	0.152	0.298	0.238
	*P*	**.001**	**.014**	**.022**	.138	**.004**	**.035**
Age	β	−0.190	−0.227	− 0.335	−0.259	− 0.128	NA
	*P*	.055	**.025**	**.001**	**.013**	.206	NA
Insall-Salvati	β	NA	NA	NA	NA	NA	−0.284
ratio	*P*	NA	NA	NA	NA	NA	**.012**
Sulcus angle	β	NA	NA	−0.246	NA	NA	NA
	*P*	NA	NA	**.008**	NA	NA	NA

Significant *P* values are bolded

*IKDC* International Knee Documentation Committee, *KOOS* Knee Injury and Osteoarthritis Outcome Score, *ADL* activities of daily living, *QoL* quality of life, *NA* not analyzed

**Fig. 1:**
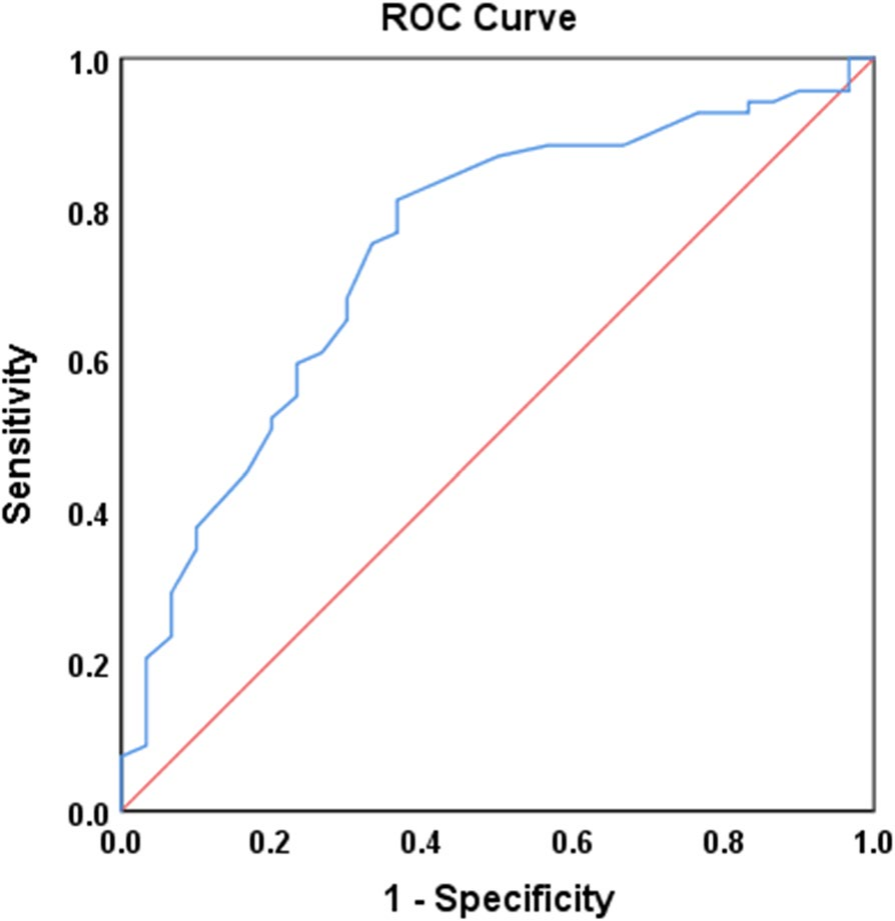
Receiver operating characteristic (ROC) curve to determine the predictive cutoff value of serum calcium for postoperative recovery based on Kujala score

In order to identify the cut-off value of serum calcium for postsurgical recovery, the ROC curve was drawn and the area under the curve was 0.744. The Youden index reached a maximum when the cutoff value of calcium was 2.225 (Fig. 1). The correspondent sensitivity was 0.812 with a specificity of 0.633. Post hoc sample size calculation showed that 86 cases were needed to achieve statistical significance with respect to specificity and 58 cases with respect to sensitivity. Given that the present cohort involved 99 patients, the acquired diagnostic information was plausible.

**Fig. 2:**
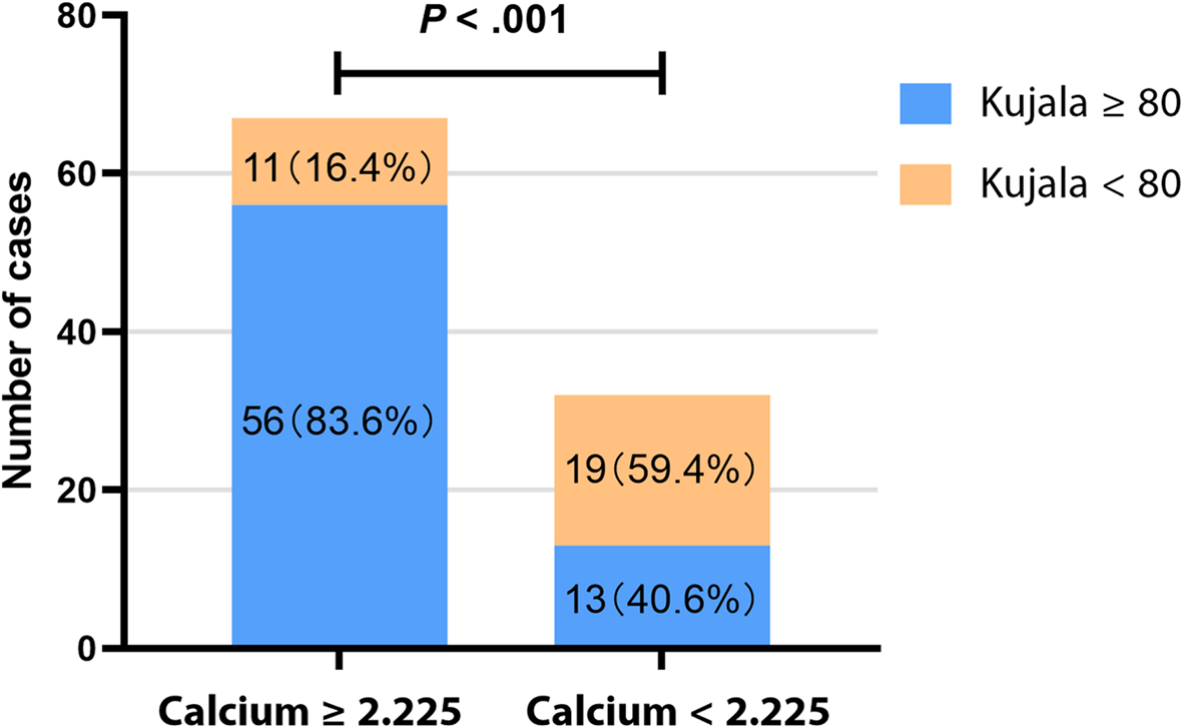
The proportion of cases with high or low Kujala score when grouped by serum calcium level

The 99 subjects were then categorized according to this cutoff value, among which 32 were in lower calcium group. The separate pre- and postoperative measurements for each group, and the comparison between the 2 groups are presented in Table 6. For cases with a higher preoperative serum calcium, the postoperative functional scores all improved significantly. In contrast, not all functional scores improved significantly in the lower calcium group and the higher calcium group had a greater score in most postoperative functional tests compared with the lower group. Additionally, by using the cutoff values of Kujala to categorize patients, it is noted that the proportion of cases with Kujala < 80 was significantly greater in cases with calcium < 2.225 mmol/L (59.4%) than those in the higher calcium group (16.4%) and the risk ratio was 3.62 (95% CI, 1.96-6.67) (Fig. 2). These results suggested that the current threshold value of calcium had practicability indeed. For rate of re-dislocation, the higher group was 3.0% (2 of 67) and the lower group was 3.1% (1 of 32). There was no significant difference between the two groups.

**Table 6:** Comparison of functional scores between the 2 groups

	Calcium ≥2.225 (*n* = 67)	Calcium < 2.225 (*n* = 32)	*P* Value
Kujala score			
Preoperative	67.5 ± 19.3	66.6 ± 21.6	.824
Final Follow-up	88.7 ± 12.2	78.1 ± 15.9	**< .001**
	***P*** **< .001**	***P*** **= .002**	
Lysholm score			
Preoperative	66.0 ± 17.8	71.8 ± 16.4	.119
Final Follow-up	86.2 ± 11.5	84.1 ± 14.8	.444
	***P*** **< .001**	***P*** **= .001**	
Tegner score			
Preoperative	2.43 ± 0.97	2.28 ± 0.89	.458
Final Follow-up	4.25 ± 1.58	3.48 ± 1.42	**.035**
	***P*** **< .001**	***P*** **= .001**	
IKDC score			
Preoperative	60.4 ± 15.2	62.0 ± 17.4	.633
Final Follow-up	77.6 ± 10.9	69.0 ± 12.8	**.001**
	***P*** **< .001**	***P*** **= .047**	
KOOS Scores			
Pain			
Preoperative	79.0 ± 15.5	75.7 ± 14.0	.317
Final Follow-up	94.5 ± 6.9	88.3 ± 10.5	**.004**
	***P*** **< .001**	***P*** **= .001**	
Symptoms			
Preoperative	59.4 ± 15.0	64.3 ± 14.0	.132
Final Follow-up	69.4 ± 15.9	72.1 ± 17.2	.444
	***P*** **= .001**	*P* = .068	
ADL			
Preoperative	77.7 ± 16.6	76.2 ± 18.9	.681
Final Follow-up	97.1 ± 4.5	93.0 ± 7.3	**.006**
	***P*** **< .001**	***P*** **< .001**	
Sports			
Preoperative	49.0 ± 28.7	58.4 ± 31.6	.140
Final Follow-up	85.1 ± 16.5	71.3 ± 19.6	**< .001**
	***P*** **< .001**	***P*** **= .039**	
QoL			
Preoperative	41.6 ± 22.4	49.4 ± 19.6	.100
Final Follow-up	66.8 ± 19.9	54.5 ± 22.6	**.011**
	***P*** **< .001**	*P* = .202	

Data are presented as mean ± standard deviation. Significant *P* values are bolded

*IKDC* International Knee Documentation Committee, *KOOS* Knee Injury and Osteoarthritis Outcome Score, *ADL* activities of daily living, *QoL* quality of life

## Discussion

In the present study, it is noted that for recurrent patellar dislocation, preoperative serum calcium level was an important factor associated with the prognosis of surgical treatment for RPD. Patients with a preoperative calcium < 2.225 mmol/L should be given extra attention postoperatively for a 3.62 times higher risk of poor recovery.

As a common issue in sports medicine, much attention is paid to RPD and numerous researchers have studied the prognostic factors. Ling et al. [6] developed a multivariable model which can identify the first-time dislocation patients who are at high risk for recurrent dislocation and thus could benefit from early surgical treatment. Age, skeletal immaturity and other anatomical factors have been identified as risk factors. Zhang et al. [19] noted that a pre-operative grade 3 J-sign was an adverse factor in operative treatment for RPD. In this study, we attempted to explore relevant factors from easily omitted but abundant blood sample data, and identified calcium as a serum biomarker for the prognosis of RPD for the first time.

As a powerful second messenger, the silent, sub-clinical variation in serum calcium is suggested to be consequential and has implications for disease susceptibility or prognosis [20–22]. It has been reported to be associated with the prognosis of various diseases, including neonatal sepsis [23], nasopharyngeal carcinoma [24], acute pulmonary embolism [16] and cognitive impairment [21]. In the present study, serum calcium level was an influential factor associated with postoperative knee function. The patients with poor postoperative recovery (Kujala < 80) had a lower preoperative calcium value than their counterparts in the study. These findings suggest that calcium level has a predictive potential for knee function and recovery after operative treatment of RPD. Patients with a preoperative calcium < 2.225 mmol/L may have a higher risk for poor recovery and low knee function. Extra attention should be paid to these patients during the rehabilitation process and the rehabilitation protocol could be individualized to better fit their recovery situation and improve their knee function.

As the most plentiful mineral in the human body, serum calcium level is usually within the normal range and affected by several vital factors, including vitamin D, parathyroid hormone, phosphorous and magnesium [25, 26]. Parathyroid hormone and vitamin D could raise blood calcium level via increasing the absorption of calcium in the intestine [25]. Increased phosphorus could bind to calcium, thereby lowering the calcium level. Low magnesium level may inhibit the release of parathyroid hormone, leading to calcium deficiency [26].

Although the exact pathophysiological mechanism underlying these clinical observations remains unclear, especially whether serum calcium level exerts a principal effect on recovery, or if it reflects a secondary epiphenomenon of other unidentified factors, there might be several possible explanations for this association. First, calcium is associated with bone formation and metabolism. Extracellular or exogenous calcium could induce mesenchymal stem cell recruitment, osteoblast differentiation and promote bone tissue regeneration [27, 28]. The healing of TT osteotomy site and bone tunnels could potentially benefit from high normocalcemia. Second, as an essential element, calcium is a crucial regulator of epidermal homeostasis and associated with cardiovascular system [29–31]. Extracellular calcium could improve skin wound repair, vascular smooth muscle cell proliferation and arterial contractility through Ca^2+^-sensing receptor, thus benefiting the postoperative recovery. Due to its role in a wide range of physiological processes, including cell signaling, neurotransmission, and muscle contraction, many other factors such as vitamin D or parathyroid hormone may underlie these clinical observations and could be studied further in the future.

Calcium, as a regulated target, is closely related to vitamin D. The intestinal absorption of calcium and phosphorus is optimized by vitamin D to formulate the bone mineral matrix [32, 33]. Calcium and vitamin D both play a crucial role in bone remodeling and mineralization, thus may impacting the orthopaedic outcomes [32]. Recently, vitamin D has been identified as an influential factor in various orthopaedic procedures. Choi et al. demonstrated that patients with vitamin D deficiency had less favorable functional outcomes after high tibial osteotomy surgery [34]. Shin et al. reported that vitamin D deficiency could adversely affect early post-operative functional outcomes following total knee arthroplasty [35]. While, the serum calcium level in their studies showed no significant difference between the vitamin D deficient and sufficient group. Due to the relatively scarce researches, the relationships between calcium, vitamin D and orthopaedic outcomes awaits investigation.

Additionally, in the present study, an older age was associated with a poor outcome in most functional scores and acted together with calcium in influencing postoperative Kujala and KOOS pain score. In line with the current study, Sambeeck et al. [36] indicated that age was correlated with an increased pain score at rest. Ling et al. [6] noted age as a variable of biggest relative importance in a multivariable model predicting for a recurrent dislocation. Since the peak incidence of first patellar dislocation is in adolescence [37], a possible explanation may be that older patients usually have a longer history of patellofemoral instability and patellar maltracking. Therefore, increased cartilage damage and degenerative changes would be seen at time of surgery, thus affecting the postoperative outcomes.

The present study has several limitations. First, patients enrolled all received TT transfer combined with MPFL reconstruction by tunnel technique, therefore the practicability of the current calcium threshold value in other surgical conditions requires more validation. Second, we could only analyze the correlation between serum calcium and the prognosis of RPD instead of disentangling the causality. Thus, we cannot determine the specific role that serum calcium plays in the pathophysiology of RPD. Third, due to the nature of retrospective study, only preoperative serum calcium was evaluated and a potential change in postoperative calcium levels and the impact on outcomes could not be evaluated. Due to the relatively scant information on related topic, it remains unknown whether a patient with preoperative low normocalcemia or hypocalcemia should receive calcium supplementation preoperatively or postoperatively. Fourth, some other contributing factors such as preoperative cartilage status, J-sign, MPFL tear pattern, generalized joint hyperlaxity and limb alignment, were not examined in this study which could add bias.

## Conclusion

Overall, operative treatment for RPD achieved good results, while in some cases the functional scores remain inferior. As a serum biomarker, preoperative calcium could be prognostic for outcomes after surgical treatment for RPD.

## Data Availability

The datasets used and/or analysed during the current study are available from the corresponding author on reasonable request.
